# Activation of distinct antiviral T-cell immunity: A comparison of bi- and trispecific T-cell engager antibodies with a chimeric antigen receptor targeting HBV envelope proteins

**DOI:** 10.3389/fimmu.2022.1029214

**Published:** 2022-11-03

**Authors:** Bilge Debelec-Butuner, Oliver Quitt, Sophia Schreiber, Frank Momburg, Karin Wisskirchen, Ulrike Protzer

**Affiliations:** ^1^ Institute of Virology, School of Medicine, Technical University of Munich/Helmholtz Centre Munich, Munich, Germany; ^2^ Department of Pharmaceutical Biotechnology, Faculty of Pharmacy, Ege University, Izmir, Turkey; ^3^ Antigen Presentation and T/NK Cell Activation Group, Clinical Cooperation Unit Applied Tumor Immunity, German Cancer Research Centre, Heidelberg, Germany; ^4^ German Centre for Infection Research (DZIF), Munich partner site, Munich, Germany

**Keywords:** HBV cure, adoptive T-cell therapy, T-cell engager antibody, drug development, chronic hepatitis B, immunotherapy, chimeric antigen receptor

## Abstract

Despite the availability of an effective prophylactic vaccine, 820,000 people die annually of hepatitis B virus (HBV)-related liver disease according to WHO. Since current antiviral therapies do not provide a curative treatment for the 296 million HBV carriers around the globe, novel strategies to cure HBV are urgently needed. A promising approach is the redirection of T cells towards HBV-infected hepatocytes employing chimeric antigen receptors or T-cell engager antibodies. We recently described the effective redirection of T cells employing a second-generation chimeric antigen receptor directed against the envelope protein of hepatitis B virus on the surface of infected cells (S-CAR) as well as bispecific antibodies that engage CD3 or CD28 on T cells employing the identical HBV envelope protein (HBVenv) binder. In this study, we added a trispecific antibody comprising all three moieties to the tool-box. Cytotoxic and non-cytolytic antiviral activities of these bi- and trispecific T-cell engager antibodies were assessed in co-cultures of human PBMC with HBV-positive hepatoma cells, and compared to that of S-CAR-grafted T cells. Activation of T cells *via* the S-CAR or by either a combination of the CD3- and CD28-targeting bispecific antibodies or the trispecific antibody allowed for specific elimination of HBV-positive target cells. While S-CAR-grafted effector T cells displayed faster killing kinetics, combinatory treatment with the bispecific antibodies or single treatment with the trispecific antibody was associated with a more pronounced cytokine release. Clearance of viral antigens and elimination of the HBV persistence form, the covalently closed circular (ccc) DNA, through cytolytic as well as cytokine-mediated activity was observed in all three settings with the combination of bispecific antibodies showing the strongest non-cytolytic, cytokine-mediated antiviral effect. Taken together, we demonstrate that bi- and trispecific T-cell engager antibodies can serve as a potent, off-the-shelf alternative to S-CAR-grafted T cells to cure HBV.

## Introduction

Chronic hepatitis B (CHB) is a global health problem affecting 296 million people (1). Direct effects of viral proteins as well as integration of HBV-DNA into the host genome induce oncogenic transformation of hepatocytes and ultimately the development of hepatocellular carcinoma (HCC) (2). HBV-related cancer is diagnosed in 30-50% of patients (3) and is the major cause for the 820,000 HBV-related deaths that are registered annually (1), clearly underlining the importance of a curative HBV therapy. Nucleos(t)ide analogues, which are the gold standard therapy for CHB, efficiently suppress HBV replication, yet, fail to achieve viral elimination, since the nuclear persistence form of HBV, the cccDNA, is not targeted (4, 5). Therefore, patients require life-long treatment and still bear a significantly increased risk to develop HBV-associated HCC (4, 5), even in the absence of obvious liver disease (6). Sustained viral control can be achieved upon interferon α-treatment; however, this regimen is only effective in 5-7% of patients and is associated with severe side effects (7, 8). New antivirals that are currently in clinical development (8, 9), such as capsid assembly modulators, entry inhibitors or siRNAs, also fail to directly target cccDNA resulting in viral relapse after cessation of therapy (10).

Cure of HBV infection will most likely require an effective strategy to activate immune responses to eliminate infected hepatocytes, induce cytokine-mediated control of HBV replication (11), and purge the cccDNA from the nucleus of infected hepatocytes by cell-intrinsic defense mechanisms (12), or at least help to permanently silence cccDNA. An ideal therapy would achieve this in combination to avoid extensive liver damage. HBV control and finally cure can be achieved by the induction of HBV-specific, polyclonal CD4^+^ and especially CD8^+^ T-cell responses, which have been established as key factors for sustained viral control, but are absent in CHB patients (13). Thus, novel immunotherapeutic approaches focus on the generation of effective T-cell responses and encouraging results have recently been published for therapeutic vaccination (14, 15), adoptive T-cell therapy (16–18) and the administration of bispecific T-cell engager antibodies (19, 20). Engineered T cells expressing HBV-specific TCRs (21–25) or CARs (16, 17, 26) as a transgene have been shown to recognize and efficiently eliminate HBV-positive hepatocytes *in vitro* and *in vivo* (27). In a similar fashion, bispecific antibodies recruit effector T cells to target cells by combining and activating CD3 and CD28 on T cells and HBVenv exposed on the surface of infected hepatocytes (19).

Current studies drive further development of both, the CAR-/TCR-T-cell technology, as well as antibody-mediated T-cell recruitment, in order to define the optimal approach for clinical application. In contrast to physiological receptors, CARs and T-cell engager antibodies can redirect T cells regardless of the patient’s MHC haplotype, which greatly broadens their applicability in the clinical setting (28, 29). To elucidate distinct characteristics of these immunotherapeutic approaches, we analyzed the efficacy of the S-CAR (16, 17) and bispecific T-cell engager antibodies (19) employing the identical HBVenv binder. To expand our T-cell engager platform and to ease therapeutic application, we used the identical HBVenv binder and constructed a trispecific T-cell engager antibody that binds HBV-infected cells, while providing stimulation as well as co-stimulation by simultaneously targeting CD3 and CD28. We comparatively studied the characteristics of these immunotherapeutic approaches concerning the activation of T cells, elimination of infected hepatocytes, as well as the eradication of HBV cccDNA to pave their path into the clinic.

## Materials and methods

### Cell culture

Huh7 and Huh7S (19) (stably transfected to express the small HBVenv protein) cell lines were cultured in Dulbecco`s minimum essential medium (DMEM) supplemented with 10% fetal calf serum (FCS), L-glutamine (2 mM), 1% non-essential amino acids and sodium pyruvate, penicillin (100 U/mL) and streptomycin (100 µg/mL) (ThermoFisher Scientific) and medium was changed every 3 days. For selection, Huh7S were cultured in the presence of 10 μg/mL blasticidin. HepG2-NTCP-K7 cells (30) stably transfected to express the Na^+^-taurocholate co-transporting polypeptide (NTCP), which has been identified as essential HBV receptor, were cultured with including the same supplements on collagen-coated plates. HepaRG cell culture and differentiation was performed as described previously (31).

### Stimulation and retroviral transduction of human T cells

Peripheral blood mononuclear cells (PBMC) were isolated *via* Pancoll gradient (Pan Biotech) according to the manufacturers protocol. For retroviral transduction, T cells were activated using Dynabeads from the Human T-Activator CD3/CD28 for T-Cell Expansion and Activation Kit (ThermoFisher Scientific) according to the provided protocol. Cells were expanded in the presence of 300 IU/mL IL-2 (Clinigen). Retroviral supernatant of S-CAR equivalent to a multiplicity of infection (MOI) of 20 infectious units (IFU)/cell was added to tissue culture plates that were coated with 20 μg/ml Retronectin (Takara), and centrifuged at 2000 x g, 32°C for 2 hours. The retroviral supernatant was removed and T cells were spinoculated on the virus-coated plate at 1000 x g for 10 min. A second transduction was performed after 24 hours. Flow cytometry analysis to quantify the percentage of transduced T cells was performed labeling cells with anti-CD4 (Biolegend), anti-CD8 (BD), and anti-IgG1 (CAR) (ThermoFischer Scientific), as well as propidium iodide (PI) or near-infrared (NIR) viability dyes (both ThermoFisher Scientific). Cells were analyzed on a CytoFlexS (Beckman Coulter) and data was processed with the FlowJo software. T cells with a transduction rate of over 95% were used for co-culture experiments.

### Co-culture with Huh7/7S cells

One day prior to co-culture with T cells or PBMC, Huh7 or Huh7S cells (4x10^4^ cells/well) were seeded in 96-well E-plates (ACEA Biosciences) to quantify cell viability, or in a conventional 96-well cell culture plate (TPP) to measure cytokine secretion, using 200 µl of culture medium/well. After removing 100 μl of medium, either PBMC combined with the indicated antibodies or S-CAR transduced T cells were added onto target cells in 100 μl at the indicated effector to target (E:T) ratio. Cell culture medium was supplemented with 10% FCS, L-glutamine (2 mM), 1% non-essential amino acids and sodium pyruvate, penicillin (100 U/mL), streptomycin (100 mg/mL), HEPES (10 mM), and gentamycin (16,6 mg/ml) (all from ThermoFisher Scientific). medium at indicated effector to target (E:T) ratios. Molarity given refers to the total antibody concentration used. The co-culture was maintained for 120 hours and cell viability was measured employing an xCELLigence RTCA SP device (ACEA Biosciences).

### Analysis of cytokine secretion by enzyme-linked immunosorbent assay

Supernatants of cell cultures were collected at indicated time points and stored at -20°C until further use. The human IFNγ (Invitrogen), human IL-2 uncoated ELISA kits (ThermoFisher Scientific) or the human TNFα ELISA set (BD OptEIA) were used according to the manufacturer’s protocol using MaxiSorp plates (ThermoFisher scientific).

### Co-culture with infected HepG2-NTCP cells

HepG2-NTCP-K7 cells (1.2x10^6^/well) (32) were seeded in collagen-coated 6-well plates (TPP) 3 days prior to infection. After 24 hours, 2.5% DMSO was added to the cell culture medium (DMEM, see cell culture) to differentiate the cells. After 48 hours, purified HBV (MOI 500 virions/cell) and 4% PEG 6000 (Sigma-Aldrich) were added. Controls were treated with 4% PEG only. After 16 hours, the inoculum was discarded, cells were washed with PBS twice, and further cultured in DMEM with 2.5% DMSO. Infection efficacy was analyzed by HBeAg ELISA using cell culture supernatant collected from 3-5 days post-infection (dpi). For co-culture experiments, cells were trypsinized 6 dpi and either 5x10^4^ infected cells/well were transferred to a 96-well E-plate for measuring cell viability using the xCELLigence RTCA system, or 3x10^5^ cells/well were transferred to 24-well plates for quantification of cytokine secretion, as well as viral DNA. 9 dpi, co-cultures were performed by addition of either PBMC combined with antibodies or S-CAR transduced T cells in the presence of 1% DMSO for 12 days. Fresh medium (containing antibodies for appropriate wells) was supplied every 3 days and supernatants were collected and stored at -20°C until further use. Cell culture supernatants of indicated time points were analyzed for cytokine levels and viral antigens, as well as HBeAg and HBsAg. At the end of the experiment, cellular DNA extraction was performed *via* a NucleoSpin tissue kit (Macherey-Nagel) according to the manufacturer’s protocol.

### Transwell assay

To analyze the cytokine-mediated antiviral effect of pro-inflammatory cytokines (e.g., IFNγ and TNFα) that are released upon T-cell activation, a transwell system was established employing transwell polyester membrane cell culture inserts with 0.4 μm pore size (Corning). Huh7 or Huh7S cells (1.6x10^5^/well) were seeded in the inlet and cultured overnight to reach a confluency of ~90%. After discarding the medium, cells were co-cultured with either S-CAR transduced T cells (4x10^5^/well) or PBMC (5.6x10^5^/well) coupled with antibodies for 24 hours. Subsequently, the inlets were transferred to 12-well cell culture plates, in which HBV infected HepaRG cells (MOI 200) were cultured as described previously (33). After one week of co-culture, cellular DNA of HepaRG cells was extracted *via* the NucleoSpin tissue kit (Macherey-Nagel) according to the manufacturer’s protocol, and viral antigens and cytokines in the supernatant were quantified.

### Quantification of viral parameters

HBsAg and HBeAg levels were quantified after appropriate dilution of samples using the quantitative HBsAg test on the Architect platform (Abbott Laboratories) and the HBeAg BEP III test (Diasorin), respectively. At the end of each experiment, total DNA was extracted from cell lysates. Total HBV-DNA and cccDNA levels were analyzed by quantitative real time PCR (qPCR) performed on a LightCycler^®^ 480 instrument (Roche Diagnostics) using selective primers (HBV-rcDNA; fwd:GTTGCCCGTTTGTCCTCTAATTC, rev:GGAGGGATACATAGAGGTTCCTTGA; cccDNA: fwd:GCCTATTGATTGGAAAGTATGT, rev:AGCTGAGGCGGTATCTA). To ensure specific detection of cccDNA (33), isolated intracellular DNA was subjected to T5 exonuclease digestion (New England Biolabs) prior to PCR as previously described (34).

### Statistical analysis

Statistical analyses were performed with Prism 9.4.1.681 (GraphPad). Data sets were analyzed with parametric 2-tailed Student’s t-tests. p values <0.05 were considered significant. The coefficients of determination (R^2^) were calculated using the Pearson correlation. Trend lines were calculated by simple linear regression.

## Results

### Design of S-CAR and T-cell engager antibodies using an identical binder

**Figure 1:**
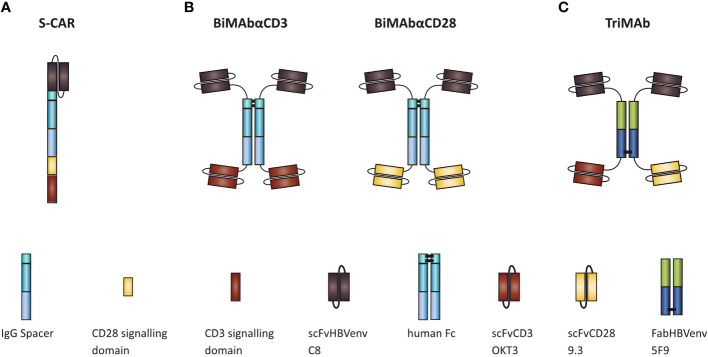
Schematic representation of **(A)** S-CAR, **(B)** BiMAb, and **(C)** TriMAb. All constructs contain the single chain fragment variable C8 for specific targeting of HBV-infected cells.

To ensure a fair comparison of S-CAR, BiMAbCD3 and BiMAbCD28 (BiMAb) and TriMAb, all constructs were equipped with the identical single chain fragment variable (scFv) C8 to specifically target the “a” determinant of HBVenv proteins (16). In the second-generation S-CAR, the C8 is linked to the cytoplasmic CD3 ζ and CD28 signaling domains by an IgG spacer and the CD28 transmembrane domain (**Figure 1A**) (16). Tetravalent BiMAb consist of two C8 and two scFv targeting either CD3 (scFv OKT3) or CD28 (scFv 9.3) for T-cell engagement (**Figure 1B**) (19). To facilitate simultaneous stimulation and co-stimulation by a single T-cell engager antibody, the HBVenv-specific Fab fragment 5F9 (19) was employed to induce heterodimerization and scFvC8 as well as scFv OKT3 and 9.3 were fused to N- and C-termini, respectively, resulting in a pentavalent construct with three binding sites for HBVenv and one binding site each for CD3 and CD28 (**Figure 1C**). In order to compare the efficiencies of S-CAR-grafted and antibody-redirected T cells, PBMC were either used in combination with T-cell engager antibodies or subjected to retroviral transduction with S-CAR after T-cell activation. A series of experiments was performed to comparatively study their ability to activate T-cell effector function in the presence of antigen, to eliminate HBVenv-expressing target cells and to elicit cytolytic and non-cytolytic antiviral effects.

### S-CAR transduced T cells eliminate HBVenv-positive target cells faster than those retargeted by T-cell engager antibodies

To study the cytotoxic potential of T cells redirected by the different means, S-CAR T cells and T cells activated in the presence of both, CD3- and CD28-activating BiMAb or TriMAb were cultured with HBVenv expressing Huh7S cells and target cell viability was quantified in real-time over 120 hours employing the xCELLigence technology. Parental Huh7 cells not expressing HBVenv and treatment with mock-transduced T cells or PBMC in the absence of engager antibodies served as controls.

**Figure 2:**
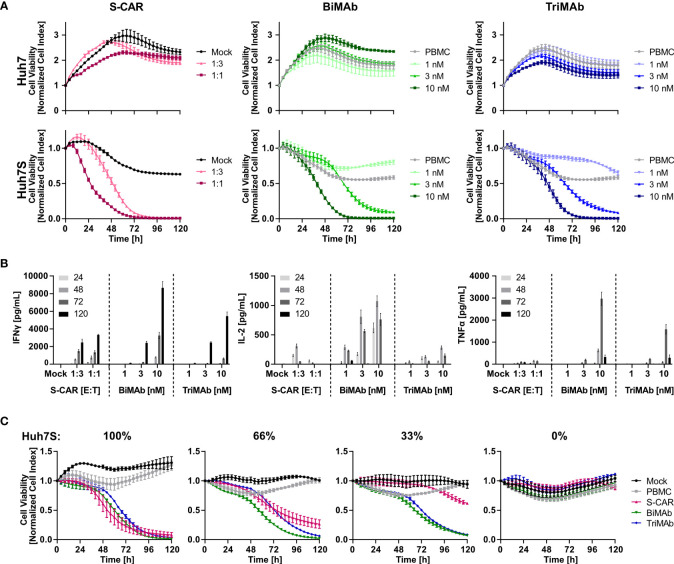
Kinetics of cytotoxicity and T-cell activation by S-CAR, BiMAb and TriMAb. S-CAR-grafted T cells at indicated E:T ratios or PBMC in combination with BiMAbαCD3 and BiMAbαCD28 in a 1:1 ratio or TriMAb at indicated concentrations were co-cultured with HBVenv-expressing Huh7S cells. Parental Huh7 cells and treatment with mock-transduced T cells or PBMC in the absence of engager antibodies served as controls. The number of PBMC was adjusted to an E:T ratio of 1:1, expecting PBMC to contain 60% T cells. **(A)** S-CAR T cells or PBMC in the presence of T-cell engager antibodies were co-cultured with Huh7 (upper panel) or Huh7S cells (lower panel) and target cell viability was determined in real-time by using an xCELLigence RTCA for 120 hours and plotted over time. **(B)** Levels of IFNγ, IL-2, and TNFα in supernatant of co-cultures collected at indicated time points (hours) were quantified by ELISA. **(C)** Cell viability of different ratios of Huh7S cells mixed with parental Huh7 cells that were co-cultured with either S-CAR T cells at an E:T of 1:1 or antibody-treated PBMC employing 10 nM of TriMAb or CD3 and CD28 BiMAb in combination. Data represent mean values ± SD of triplicates (n = 3).

Cell viability decreased specifically in a dose-dependent manner in all three settings leading to complete elimination of target cells within 72 hours using an E:T ratio of 1:1 for S-CAR T cells or 10 nM of antibody in combination with PBMC. S-CAR-transduced T cells were able to eliminate HBVenv-expressing target cells fast and specifically, resulting in a pronounced cytotoxic effect within 24 hours with Huh7 control cells remaining fully viable over the course of the experiment (**Figure 2A**). BiMAb and TriMAb treatment also showed a high specificity, but significant cytotoxic effects were only observed after 24 hours and showed slower kinetics. Full cytotoxicity required a >3 nM concentration of either antibody (**Figure 2A**).

To study T-cell activation, cell culture supernatants were collected after 24, 48, 72 and 120 hours and levels of IFNγ, IL-2 and TNFα were quantified by ELISA. Cytokine levels correlated with the E:T ratio of S-CAR-transduced cells and the concentration of antibodies applied. Interestingly, BiMAb/TriMAb-activated PBMC released higher cytokine levels than S-CAR-transduced T cells. The cytokine secretion kinetics were identical in all three settings with IL-2 being released early on with a peak after 48 hours, while TNFα and IFNγ were detected later with peaks at 72 and 120 hours, respectively (**Figure 2B**).

To analyze specificity and activation thresholds of T-cell redirection in more detail, mixtures of Huh7S with parental Huh7 cells from 0% to 100% were co-cultured with the respective effector cells over 120 hours. In the presence of S-CAR T cells, the number of eliminated hepatoma cells correlated well with the percentage of HBVenv-expressing target cells indicating a high specificity of the cytolytic activity despite the secretion of HBsAg. In contrast, T-cell engager antibodies induced a sustained loss of cell viability, also if only 66% or 33% of Huh7S cells were present, while in their absence cell viability of parental Huh7 cells was not affected (**Figure 2C**). This suggested that activation of T cells upon treatment with T-cell engager antibodies is dependent on the presence of HBV envelope protein, but the HBsAg-producing cells can induce a potent T-cell activation resulting in collateral damage of HBV-negative Huh7 cells either due to the extremely high cytokine levels produced by the activated T cells or due to binding of secreted HBsAg.

### Specific elimination of HBV-infected hepatocytes correlates with a potent antiviral effect.

**Figure 3:**
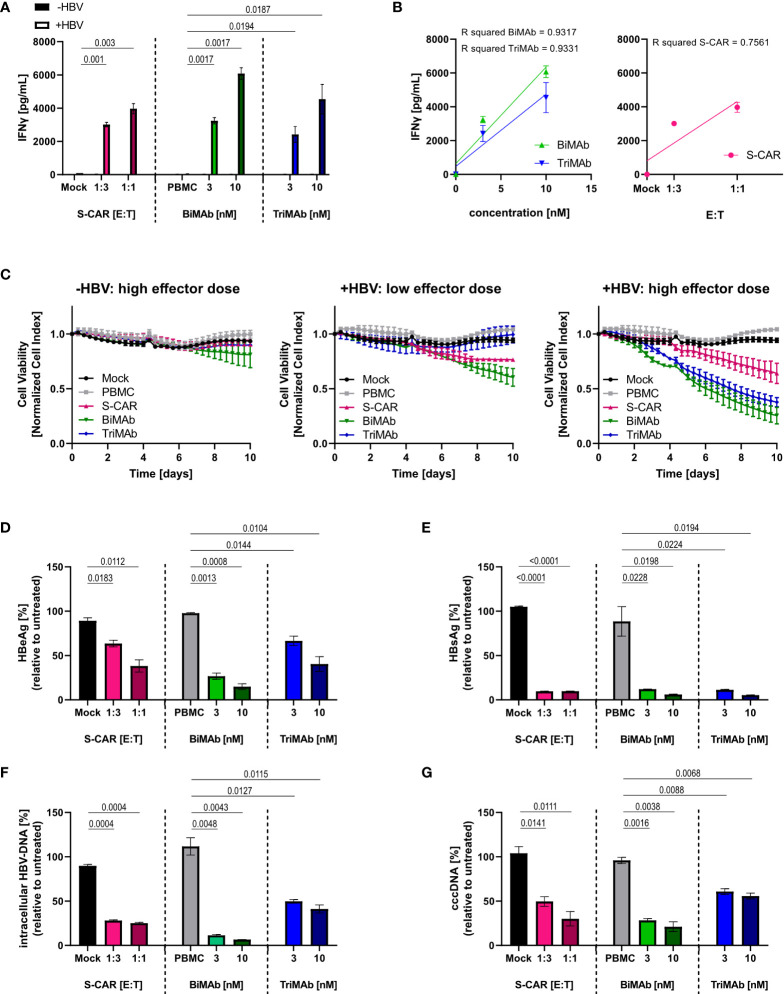
Dose dependency, specificity and antiviral effect of T cells activated via S-CAR, BiMAb and TriMAb. HepG2-NTCP-K7 cells were infected at an MOI of 500 virions/cell (+HBV) and co-cultured for 12 days with either S-CAR-grafted T cells at at E:T ratios of 1:3 (low effector dose) or 1:1 (high effector dose) or PBMC in combination with BiMAbαCD3 and BiMAbαCD28 in a 1:1 ratio or TriMAb at the indicated concentrations (3 nM = low dose, 10 nM = high dose). Uninfected HepG2 cells (-HBV) and treatment with mock-transduced T cells or with PBMC in the absence of engager antibodies served as controls. The number of PBMC was adjusted to an E:T ratio of 1:1, expecting PBMC to contain 60% T cells. **(A)** The IFNγ concentration in the supernatant of co-cultures on day 3 was quantified by ELISA. **(B)** The coefficient of determination (R^2^) between IFNγ levels in the supernatant and antibody concentration (left) or E:T ratio (right) was calculated using the Pearson correlation. Trend lines were calculated by simple linear regression. **(C)** The viability of uninfected controls (left), infected HepG2 cells treated with low effector dose (middle), or high effector dose (right) was determined over 10 days employing an xCELLigence real-time cell analyzer and plotted over time. **(A–C)** Data are presented as mean values ± SD of triplicates (n = 3). Levels of **(D)** HBeAg and **(E)** HBsAg in the supernatant produced from day 9-12. **(F)** Intracellular HBV-DNA and **(G)** cccDNA in cell lysates were quantified by qPCR after 12 days of co-culture. **(D–G)** Results are presented as percentage relative to untreated controls. Data are presented as mean values ± SD of duplicates (n = 2). To calculate p values, we employed 2-tailed unpaired t-tests.

To characterize the elimination of HBV-infected cells and quantify antiviral effects, HepG2-NTCP-K7 cells were infected at an MOI of 500 HBV virions/cell and treated with S-CAR T cells or PBMC in combination with BiMAb or TriMAb. Uninfected HepG2-NTCP cells and treatment with mock-transduced T cells or PBMC in the absence of engager antibodies served as controls. Activation of T cells was detected by secretion of IFNγ into the cell culture supernatant at day 3 exclusively in co-cultures with HBV-infected cells proving antigen-dependent T-cell activation. At a high dose (10 nM), the BiMAb combination resulted in maximal IFNγ release (**Figure 3A**). IFNγ levels correlated with the E:T ratio, as well as the antibody concentration (**Figure 3B**). Quantification of cell viability revealed no effect on uninfected controls, while infected HepG2 cells were eliminated in an E:T ratio- and dose-dependent manner with T-cell engager antibodies exhibiting an enhanced cytolytic activity compared to S-CAR T cells (**Figure 3C**).

To quantify the antiviral effect, several parameters determining HBV replication were analyzed after 12 days of co-culture. A significant reduction of HBeAg and HBsAg in the supernatant, as well as intracellular HBV DNA and cccDNA was observed in all three settings showing a tendency towards a stronger effect upon treatment with BiMAb antibodies compared to S-CAR T cells and TriMAb (**Figures 3D–G**). Interestingly, no clear dose dependency of the antiviral effect was observed, while killing kinetics were dose-dependent. This led to the assumption that the activated T cells elicited not only cytolytic but also non-cytolytic, most likely cytokine-mediated antiviral effects.

### Treatment with T-cell engager antibodies mediates a robust cytokine-mediated antiviral effect

Since it is known that T cells contribute to control of HBV by non-cytolytic mechanisms (12, 35), we raised the question whether the elevated cytokine levels upon T-cell engager treatment would result in an increased antiviral effect of BiMAb compared to S-CAR T cells. To quantify the cytokine-mediated antiviral effect independent of T-cell cytotoxicity, Huh7S cells were cultured together with T cells activated *via* the S-CAR or by T-cell engager antibodies in a transwell system for 7 days and in the same well, but physically separated from HBV-infected, differentiated HepaRG cells. Differentiated HepaRG cells were chosen, because, in contrast to most hepatoma cell lines, they still have intact cytokine signaling pathways and closely resemble primary human hepatocyte cultures as described previously (19, 33). In this setup, effector cells are activated by HBVenv-expressing Huh7S cells in the transwell, while direct contact to HBV-infected HepaRG cells on the bottom of the plate and direct killing of infected cells is prevented. Thus, antiviral activity is exclusively mediated by soluble factors.

**Figure 4:**
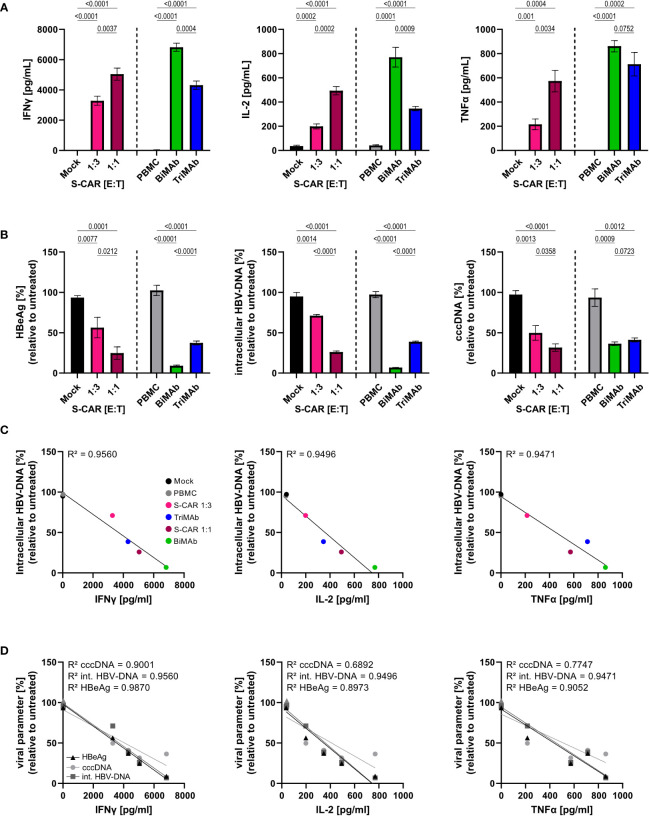
Non-cytolytic antiviral effect of S-CAR T cells and PBMC activated by T-cell engager antibodies. HepaRG cells were differentiated and infected with HBV at an MOI of 200 virions/cell. Huh7S cells and effector cells were cultured in a transwell inserted into wells containing HBV-infected HepaRG cells to avoid direct contact. In the inlet, S-CAR-grafted T cells at indicated E:T ratios or PBMC in combination with 10 nM BiMAbαCD3 and BiMAbαCD28 in a 1:1 ratio, or 10 nM TriMAb were co-cultured with HBVenv-expressing Huh7S cells for 7 days. Mock-transduced T cells or PBMC in the absence of engager antibodies served as controls. The number of PBMC was adjusted to an E:T ratio of 1:1, expecting PBMC to contain 60% T cells. **(A)** Levels of IFNγ (left), IL-2, (middle) TNFα (right) and in the supernatant were quantified by ELISA. **(B)** HBeAg in the supernatant (left), or intracellular HBV-DNA (middle) and cccDNA (right) in cell lysates were quantified by HBeAg BEP III test or qPCR, respectively. **(C)** The coefficient of determination (R^2^) between the levels of intracellular HBV-DNA in lysates and the concentration of IFNγ (left), IL-2 (middle), and TNFα (right) in the supernatant was calculated using the Pearson correlation. **(D)** The coefficient of determination (R^2^) between the levels of the viral parameters HBeAg, intracellular HBV-DNA, or cccDNA, and the concentration of IFNγ (left), IL-2 (middle), and TNFα (right) in the supernatant was calculated using the Pearson correlation. Trend lines were calculated by simple linear regression. Data are presented as mean values ± SD of triplicates (n = 3). To calculate p-values, we used 2-tailed unpaired t-tests.

Quantification of cytokine levels after 7 days showed that S-CAR T cells, as well as T cells activated by T-cell engager antibodies released IFNγ, IL-2, and TNFα in the presence of HBVenv-positive Huh7S cells. Treatment with 10 nM BiMAb in combination resulted in the highest cytokine levels, followed by S-CAR-grafted T cells at an E:T of 1:1 and 10 nM TriMAb (**Figure 4A**). Assessment of viral parameters revealed an antiviral effect although the T cells had no direct contact to infected cells. High cytokine levels in BiMAb-treated cultures resulted in over 90% reduction of intracellular HBV-DNA and HBeAg and at least 50% reduction of viral cccDNA was observed in all three setups (**Figure 4B**), clearly demonstrating that T cells activated either by the S-CAR or by T-cell engager antibodies elicit a significant non-cytolytic antiviral effect. The strength of this non-cytolytic antiviral effect indirectly correlated with cytokine levels, indicating that higher cytokine levels correlated with lower HBV replication markers and a stronger antiviral effect, respectively (**Figures 4C, D** **)**.

## Discussion

In this study we aimed to evaluate the potential of HBV-specific T-cell redirection by comparatively analyzing a second-generation CAR and T-cell engager antibodies. We compared the antiviral efficacy of TriMAb as a novel, trispecific T-cell engager antibody, combining activation *via* CD3 and CD28 to that of a combination of CD3- and CD28-BiMAb and the S-CAR known to activate full T-cell effector function. We demonstrated T-cell activation and strong cytotoxic and non-cytolytic antiviral effects of T cells redirected by both, bi- and trispecific T-cell engager antibodies, as well as the S-CAR in correlation with antibody concentration or E:T ratio, respectively. Hereby, the combination of the two BiMAb showed the strongest antiviral effect which was to a large part cytokine-mediated with T-cell cytokines controlling HBV replication (35) and purging HBV cccDNA from the nucleus of infected cells (12).

Chronic hepatitis B and HBV-related HCC are a global health problem and curative treatment remains elusive. Once HCC develops, patients have a poor prognosis (36) and clearance of HBV in early-stage disease seems key to reduce morbidity and mortality in infected individuals. However, approved therapies, as well as novel treatment options currently in clinical development, suppress viral replication but are unable to cure the disease as they do not target the covalently closed circular DNA (37). Clearance of cccDNA requires the elimination of all infected hepatocytes or the confined effective release of proinflammatory cytokines to induce non-hepatotoxic purging of the persistent viral DNA (12). Both seems accessible by the induction of a robust T-cell response.

Preclinical assessment of immunotherapeutic strategies provided strong evidence that the artificial generation of HBV-specific immunity can be exploited to treat HBV-related liver disease (15, 19, 27, 38). Since we have previously shown that only the combinatory treatment with HBVenv-CD3 and HBVenv-CD28 engager antibodies is able to induce an effective T-cell response when targeting HBV infected cells (19), we additionally generated a novel trispecific T-cell engager antibody and included it in this study. All three setups, the S-CAR, the BiMAb and the TriMAb induce CD3 and CD28 signaling. Varying affinity to the HBVenv target proteins were minimized by incorporation of the identical HBV binder. For specific targeting of HBVenv, we chose the binder C8, since it recognizes all HBV geno- and subtypes (19) and therefore is an optimal candidate with broad applicability in the majority of CHB patients. HBVenv proteins are exposed on HBV-infected cells (39), but are also expressed in about 30% of HBV-related tumor tissue (40), which might additionally allow specific targeting of certain HCCs.

In our experiments, S-CAR-grafted T cells were able to eliminate 50% of target cells within 24 hours, which is in line with recent studies employing CAR T cells (41, 42). In comparison to S-CAR T cells, induction of cytotoxic activity upon engager antibody treatment was delayed, resulting in a 50% elimination rate within 48 hours. Similar kinetics have been observed by *Sung* et al. using an HIV-targeting bispecific antibody (43). Faster target cell elimination by CAR-grafted T cells indicates that extracellular triggering of CD3 and CD28 *via* engager antibodies is less efficient than direct activation by intracellular signaling. However, it needs to be considered that S-CAR T cells are heavily pre-stimulated during retroviral transduction, which affects subset composition and induces proliferation and differentiation of naïve T cells (44). Under physiological conditions, naïve T cells typically undergo an expansion phase upon antigen encounter before they exert effector function (45). This first antigen encounter is mimicked by CD3/CD28 triggering during transduction, but is absent in the PBMC population. We and others have shown that naïve T cells will expand in a similar fashion upon engagement with bispecific antibodies (19, 46), providing a plausible explanation for the delayed induction of cytotoxic activity. We deliberately decided against any pre-stimulation of PBMC in our T-cell engager assays to provide a close reflection of a potential clinical setting.

Interestingly, the faster killing kinetics by S-CAR T cells was not observed in co-cultures with infected HepG2-NTCP-K7 cells indicating that the cytotoxic activity might also be modulated by additional factors, such as e.g. antigen density on the target cells. In our infection experiments, high cytokine levels upon BiMAb treatment correlated with enhanced cytotoxic activity and an improved antiviral effect. Here, the number of targets and antigen density are reduced compared to Huh7S cultures. We have previously shown that an MOI of 500 virions/cell results in an infection rate of about 60% (19). Thus, the number of targets was comparable to the mixed cultures with 66% Huh7S cells, where we also found higher cytotoxic activity in BiMAb-treated samples.

T-cell activation in all settings strictly depended on the presence of HBVenv protein and no cytokine release was observed in cultures with parental Huh7 or uninfected HepG2-NTCP cells. However, we detected an excess elimination of target cells in mixed cultures of Huh7 and Huh7S cells, indicating cytotoxic activity against antigen-negative cells upon treatment with T-cell engager antibodies. This effect is most likely mediated by the high concentrations of cytokines and granzyme B (data not shown) that we observed upon treatment, even at low target cell numbers, as e.g. TNFα is able to induce apoptosis (47). Nevertheless, T-cell activation through binding of HBsAg on negative target cells cannot be excluded. These findings indicate that treatment with T-cell engager antibodies confers enhanced sensitivity at low antigen density, but the tremendous release of proinflammatory cytokines may reduce specificity, at least in a cell culture setting. As cytokines can continuously accumulate to unphysiologically high levels in cell cultures, animal studies are required to understand if this effect will also play a role *in vivo*.

Multiple studies provide convincing evidence that antigen-dependent co-stimulation through CD28-targeting bispecific T-cell engager antibodies is an effective strategy to specifically increase T-cell activation *in vitro* (19, 48) and enhance anti-tumor activity *in vivo* (46, 49). In the context of persistent HBV infection, co-stimulation was even essential to induce significant T-cell activation and target-cell elimination (19). Even though combination therapy with CD3- and CD28-engaging bispecifics is considered for clinical application (49–51), the development of antibody cocktails is associated with tremendous pre-clinical workload and high expenses during clinical trials, since pharmacology studies must be conducted for each molecule individually, as well as in combination (50). As a therapeutic alternative, we designed a trispecific antibody that combines the specificities for HBVenv, CD3 and CD28 in a single molecule.

To avoid sequential linking of multiple scFvs, which in our case was associated with low stability and tendency to aggregation (data not shown), we decided to incorporate an efficient heterodimerization domain. IgG heavy chains are capable of forming such heterodimers in addition to homodimers, but only 33% of purified molecules will contain the correct combination. Even though advances in recombinant antibody technology led to the development of heterodimeric Fc domains (52), production of these molecules remains challenging, and IP protection limits the freedom to operate. We therefore decided to design a novel trispecific scaffold and chose a Fab fragment as heterodimerization domain. The generation of heterodimeric antibody derivates on the basis of Fab-fragments was described before (53), but has not yet been thoroughly exploited in a therapeutic setting.

Recently, the first trispecific T-cell engager antibody successfully made its way into the clinic. SAR442257 by Sanofi targets CD38, CD3 and CD28 (54) and is currently under investigation in a phase I trial for the treatment of relapsed and refractory multiple myeloma and non-Hodgkin’s lymphoma (NCT04401020). We therefore aimed at developing the TriMAb as a single-molecule alternative to the BiMAb combination. While such trispecific molecules certainly ease clinical development of a highly potent T-cell engager, they are by design restricted to a rigid ratio of stimulatory and co-stimulatory signals. Antibody cocktails, in contrast, provide a higher flexibility as they can be applied in various ratios depending on disease stage or patient background. Additionally, specificity and safety can be enhanced by targeting individual molecules with the respective CD3 and CD28 engager similar to signaling complementation strategies for CARs (55, 56). To this end, we convincingly show that TriMAb efficiently induces T-cell activation and target cell elimination, but is functionality not superior to the combination of BiMAb. It remains unclear, whether the steric arrangement of scFvs and target molecules in an immunological synapse allows for simultaneous engagement of all three antigens by a single TriMAb molecule. Additionally, TriMAb allows only monovalent binding to T-cell antigens resulting in comparatively limited T-cell activation. Increasing the number of scFvs resulted in reduced stability or antigen-independent T-cell activation, which in turn impaired specific redirection in our experiments (data not shown). Additional work is still required to enhance TriMAb efficacy, while protecting stability and specificity.

Overall, S-CAR T cells and T-cell engager antibodies showed similar efficacy in eliminating HBV-positive targets and impairing viral replication. For further clinical development, production efficacy under GMP conditions and product stability need to be considered, independent of initial efficacy. T-cell engager antibodies do not require a specialized production facility, can be used off-the-shelf, are simple to administer and additionally do not require handling or genetic modification of immune cells. However, clinical data for T-cell engager antibodies is limited, since currently only blinatumomab as effective treatment option for certain B-cell malignancies is successfully used in the clinics (57). A major challenge in clinical development of bispecific antibodies is the induction of a cytokine release syndrome and associated toxicity, which led to the termination of clinical trials of promising candidates such as duvortuxizumab or AFM11 (58). Thus, a balanced fine-tuning of T-cell activation *in vivo* and careful evaluation of dosing regimens are key factors to be considered in future clinical development. In contrast, CAR-T cells maintain the physiological T-cell regulation and silencing abilities and have successfully made their way into the clinics with numerous trials that illustrate the efficacy and safety in patients (59). This resulted in the approval of five CAR T-cell products by the FDA within the last years (60). A key feature of stably transduced cells that cannot be achieved with T-cell engagers, is specific expansion, persistence, and memory formation of the transferred cells, which has been shown to correlate with long-term tumor immunity without relapse (61). The vast knowledge on CAR T-cell therapy that is already available facilitates a rapid clinical translation of novel CAR T-cell products, as important information about efficacy and risk factors are easily accessible to authorities. Translation of T-cell engager products is comparatively challenging as it still faces a number of hurdles, but these molecules bear a great therapeutic potential that will be exploited in the near future.

Taken together, chimeric antigen receptors and bispecific, as well as trispecific T-cell engager antibodies are powerful immunotherapeutic tools that provide an interesting novel option for the treatment of chronic hepatitis B and HBV-related HCC. By comparatively evaluating the characteristics of these distinct redirection methods we provided novel insights into their functionality and support their translation into the clinic.

## Data availability statement

The raw data supporting the conclusions of this article will be made available by the authors, without undue reservation.

## Author contributions

UP, BD-B, and OQ designed the study. BD-B and OQ performed experiments and collected data. BD-B, OQ and UP analyzed the data. FM, SS and KW provided reagents, technical support and conceptual advice. OQ, BD-B and UP wrote the manuscript. All authors critically reviewed the manuscript. All authors contributed to the article and approved the submitted version.

## Funding

The study was supported by the Deutsche Forschungsgemeinschaft (DFG, German Research Foundation) – SFB-TRR 338/1 2021–452881907 and SFB-TRR 179/2 2020 –272983813 and by a research collaboration with SCG Cell Therapy. The funders had no influence on the content of the manuscript. BDB was supported by The Scientific and Technological Research Council of Turkey 2219 Postdoctoral fellowship program.

## Acknowledgments

The authors thank Theresa Asen, Romina Bester, and Philipp Hagen for excellent technical support.

## Conflict of interests

UP and FM are named as inventors on the patent WO 2015/036606 held by HMGU and DKFZ. UP, FM, and OQ are named as inventors on the patent WO 2016/146702 held by HMGU, DKFZ and TUM. UP serves as ad hoc advisor for Abbott, Aligos, Arbutus, Gilead, GSK, Merck, Sanofi, Roche and VirBiotech. UP is co-founder and share-holder of SCG Cell Therapy who licensed patents WO 2015/036606 and WO 2016/146702. KW is Managing Director/Head of Research and share-holder of SCG Cell Therapy Germany.

The remaining authors declare that the research was conducted in the absence of any commercial or financial relationships that could be construed as a potential conflict of interest.

## Publisher’s note

All claims expressed in this article are solely those of the authors and do not necessarily represent those of their affiliated organizations, or those of the publisher, the editors and the reviewers. Any product that may be evaluated in this article, or claim that may be made by its manufacturer, is not guaranteed or endorsed by the publisher.
